# Quality Improvement to Improve the Did Not Attend (DNA) and Cancellation Rates of the Opt-in Process for the Adolescent and Young Adult Service (AYAS)

**DOI:** 10.1192/bjo.2026.11501

**Published:** 2026-06-30

**Authors:** Oliver Tran, Fatma Eren, Sheva Habel, Emma Mittelman, Mariana Garcia

**Affiliations:** The Tavistock and Portman NHS Foundation Trust, London, United Kingdom

## Abstract

**Aims:**

To improve access to the young person psychotherapy service by adapting intake requirements. National data indicates that there is an impact of ethnicity on accessing therapies. Our aims are to improve attendance of the initial pre-assessment consultation (PAC) appointments, but also to see if there is an improvement to PCREF diversity. To support the AYAS team in achieving the existing 4-week KPI target between referral and appointment by reducing DNAs and cancellations.

**Methods:**

1) Data was collected during the period between April 2024–June 2025 on patients who either did not attend (DNA) or cancelled their initial pre-assessment consultation (PAC) appointments. Patient records were reviewed to understand the most common reasons why patients were unable to attend their appointments.

2) Stakeholder engagement was also sought by collecting opinions from staff members involved in the opt-in process.

3) Using the above data and feedback, a new opt-in template was developed that was user friendly, age appropriate, and helpful for booking appointments, thus improving the experience for patients and staff.

4) Following its implementation, data was collected on whether the new process was effective in achieving our initial aims and objectives. Continued feedback will be sought from patients and staff members to inform a new cycle of quality improvement in the future.

**Results:**

Data collected in the 14 months between April 2024–June 2025 found 21 DNAs and cancellations. The most common reason given for cancellations and DNAs was because their appointment clashed with college, work or other commitments (10). This was followed by being given too short notice (5) and sickness (4). Of the 304 appointments offered in 2024–2025, there were 42 first-appointment DNAs. Of these, the most common ethnicities were White British (10), White Other (6) and Black African (6). There was an improvement in the time it took for patients to respond once an opt-in was offered, which was supported by feedback from staff members involved in the opt-in process.

**Conclusion:**

The new opt-in process for the Adolescent and Young Adult Service (AYAS) has demonstrated promising results in improving the rates of response for patients offered an opt-in to attend the pre-assessment consultation (PAC). Previous data suggests that there is no impact of diversity on attendance. Ongoing data collection is required to assess its longer-term impact on the service.

